# Resolved ALMA observations of water in the inner astronomical units of the HL Tau disk

**DOI:** 10.1038/s41550-024-02207-w

**Published:** 2024-02-29

**Authors:** Stefano Facchini, Leonardo Testi, Elizabeth Humphreys, Mathieu Vander Donckt, Andrea Isella, Ramon Wrzosek, Alain Baudry, Malcom D. Gray, Anita M. S. Richards, Wouter Vlemmings

**Affiliations:** 1 Dipartimento di Fisica, Università degli Studi di Milano, Milano, Italy; 2 Dipartimento di Fisica e Astronomia ‘Augusto Righi’, Università di Bologna, Bologna, Italy; 3 European Southern Observatory, Garching bei München, Germany; 4 Joint ALMA Observatory, Santiago, Chile; 5 European Southern Observatory (ESO) Vitacura, Santiago, Chile; 6 Space sciences, Technologies & Astrophysics Research (STAR) Institute, University of Liège, Liège, Belgium; 7 Department of Physics and Astronomy, Rice University, Houston, TX USA; 8 Laboratoire d’Astrophysique de Bordeaux, Univ. de Bordeaux, CNRS, Pessac, France; 9 National Astronomical Research Institute of Thailand, Chiangmai, Thailand; 10 Jodrell Bank Centre for Astrophysics, University of Manchester, Manchester, UK; 11 Department of Space, Earth and Environment, Chalmers University of Technology, Göteborg, Sweden

**Keywords:** Astrophysical disks, Exoplanets, Early solar system

## Abstract

The water molecule is a key ingredient in the formation of planetary systems, with the water snowline being a favourable location for the growth of massive planetary cores. Here we present Atacama Large Millimeter/submillimeter Array data of the ringed protoplanetary disk orbiting the young star HL Tauri that show centrally peaked, bright emission arising from three distinct transitions of the main water isotopologue (${\mathrm{H}}_{2}^{16}{\mathrm{O}}$). The spatially and spectrally resolved water content probes gas in a thermal range down to the water sublimation temperature. Our analysis implies a stringent lower limit of 3.7 Earth oceans of water vapour available within the inner 17 astronomical units of the system. We show that our observations are limited to probing the water content in the atmosphere of the disk, due to the high dust column density and absorption, and indicate that the main water isotopologue is the best tracer to spatially resolve water vapour in protoplanetary disks.

## Abstract (web summary)

Spectrally and spatially resolved ALMA observations of water vapour in the inner regions of the famous planet-forming disk around HL Tauri pave the way towards an observational characterization of planet formation at the water snowline.

## Main

The water molecule is undoubtedly one of the most important molecular species in the whole Universe. Being an extremely efficient solvent, water had a key role in the emergence of life as we know it on our planet. For this reason, the chemical characterization of exoplanetary atmospheres is often focused on detecting this particular molecular species^1–3^. Formed by the common H and O atoms, water plays a fundamental role in the physics of the formation of planetary systems, due to its very high abundance in both gaseous and icy forms^4,5^. Theoretical models predict that at the location of the phase transition from gaseous to solid form, dust grains can accumulate and grow very efficiently, promoting the fast formation of planetary cores. Across this particular radial location, called the ‘snowline’, grains can drastically change their drift and fragmentation velocity, composition and opacity. In synergy with vapour radial diffusion^6^, these physical discontinuities can lead to the accumulation and growth of dust grains into planetesimals^7,8^. The position of the snowline also defines the chemistry of the available planet building blocks. Since the H_2_O molecule is the major elemental oxygen carrier in the disk, its desorption and freezing affect the elemental C/O ratio in both the gas and solid phases^9–11^.

Because of its large binding energy, the H_2_O transition from ice to gas happens a few astronomical units (au) from the young star where the midplane temperatures are in the range from 100 to 200 K, making it the last major ice component to sublimate. However, the proximity to the host star makes the detection of the snowline complicated even in the closest star-forming regions. Both cold and warm water lines have been detected in a few disks by Herschel (see refs. ^12,13^ and references therein), Spitzer^14^, JWST^15–17^ and ground-based observatories^18^, but the low angular resolution did not allow robust inferences about the extent of the water snowline. Directly observing water emission from the ground is complicated by the high water vapour content of the Earth’s atmosphere, resulting in strong telluric absorption. To circumvent this problem, most programmes at millimetre wavelengths have focused on attempting the detection of the rarer ${\mathrm{H}}_{2}^{18}{\mathrm{O}}$ and HDO isotopologues, leading to the clear detection of spatially resolved water isotopologue emission in the outbursting source V883 Ori (ref. ^19^). In quiescent sources, except the candidate detections in the AS 205N and HL Tauri (HL Tau) disks, no detection of thermal emission at (submillimetre) wavelengths has been reported in the literature^20–23^.

In this Article, we focus on the textbook case of HL Tau, the first protoplanetary disk imaged at very high angular resolution (~0.025*″*) with the Atacama Large Millimeter/submillimeter Array (ALMA)^24^. The disk shows a spectacular pattern of concentric rings. With the source being young (≲1 Myr) and the dynamical stellar mass being relatively high (2.1 ± 0.2 *M*_⊙_; ref. ^25^), the inner disk temperatures are expected to be warm, due to irradiation and accretion heating. Warm and hot water has been detected in HL Tau both by Herschel in the far infrared^26^ and by ground-based high-resolution spectroscopy in the mid-infrared^27^, with the lines not being spatially resolved. With this Article, we report the detection of three rotational water lines in the inner regions of the HL Tau protoplanetary disk obtained with ALMA. The lines are spectrally resolved. Analysis of the interferometric data confirms that the extent of the water emission is confined within a prominent gap seen in the HL Tau continuum intensity. These data present spatially resolved images of the emission from the main water isotopologue (H_2_O) in a protoplanetary disk and pave the way to a new observational strategy to characterize the water vapour content of terrestrial planet-forming regions.

## Observations

We observed HL Tau in two different ALMA bands (band 5, originally developed with the goal of studying water in the local Universe^28^, and band 7) to target three transitions of water (two lines of para-water and one line of ortho-water): p-H_2_O 3_13_–2_20_ and 5_15_–4_22_, at 183.31 and 325.15 GHz, respectively, and o-H_2_O 10_29_–9_36_ at 321.22 GHz. The first two lines are expected to trace warm water vapour outside the water snowline, while the third line is predicted to detect hot water within the water snowline^29,30^. We also observed a rotational transition of the water isotopologue p-${\mathrm{H}}_{2}^{18}{\mathrm{O}}$ at 322.46 GHz. The molecular coefficients of the lines and sensitivity of the observations are reported in Tables 1 and 2.

**Table 1:** Observed H_2_O isotopologue transitions

Transition	Rest Frequency *ν*_0_	*E*_u_	Channel width	r.m.s.^a^	Flux	Mask radius^b^
	(GHz)	(K)	(km s^−1^)	(mJy bm^−1^)	(mJy km s^−1^)	(″)
p-H_2_O 3_13_–2_20_	183.31009	204.7	0.8	6.90	973 ± 89	0.70
p-H_2_O 5_15_–4_22_	325.15290	469.9	1.0	3.71	1332 ± 89^c^	0.70
o-H_2_O 10_29_–9_36_	321.22569	1,861.3	5.0	0.96	679 ± 135	0.28
p-${\mathrm{H}}_{2}^{18}{\mathrm{O}}$ 5_15_–4_22_	322.46517	467.9	1.0	1.75	<33.6^d^	0.70

The upper state energies are taken from ref. ^51^. The notation for the water energy levels in the vibrational ground state is ${J}_{{K}_{a},{K}_{c}}$. ^a^Obtained over one single channel. ^b^Circular radius used to extract the line flux. ^c^This flux measurement corrects for the absorption identified at ~10 km s^−1^. The flux obtained without accounting for absorption is 929 ± 89 mJy km s^−1^. ^d^3*σ* upper limit.

**Table 2:** Observation IDs and execution block properties, including source integration time, median PWV column, bandpass, flux and phase calibrators and maximum baseline

Program ID	Lines	Integration time (min)	PWV (mm)	Bandpass/flux calibrators	Phase calibrators	Maximum baseline (m)
2017.1.01178.S	p-H_2_O 3_13_–2_20_	46	0.2	J0423-0120	J0510+1800	1,398
2017.1.01178.S	p-H_2_O 5_15_–4_22_	31	0.4	J0538-4405	J0431+1731	3,637
2017.1.01178.S	o-H_2_O 10_29_–9_36_	33	0.5	J0519-4546	J0440+1437	8,547
2022.1.00905.S	p-H_2_O 5_15_–4_22_	100	0.3	J0423-0120	J0431+1731	500
	p-${\mathrm{H}}_{2}^{18}{\mathrm{O}}$ 5_15_–4_22_					

**Fig. 1:**
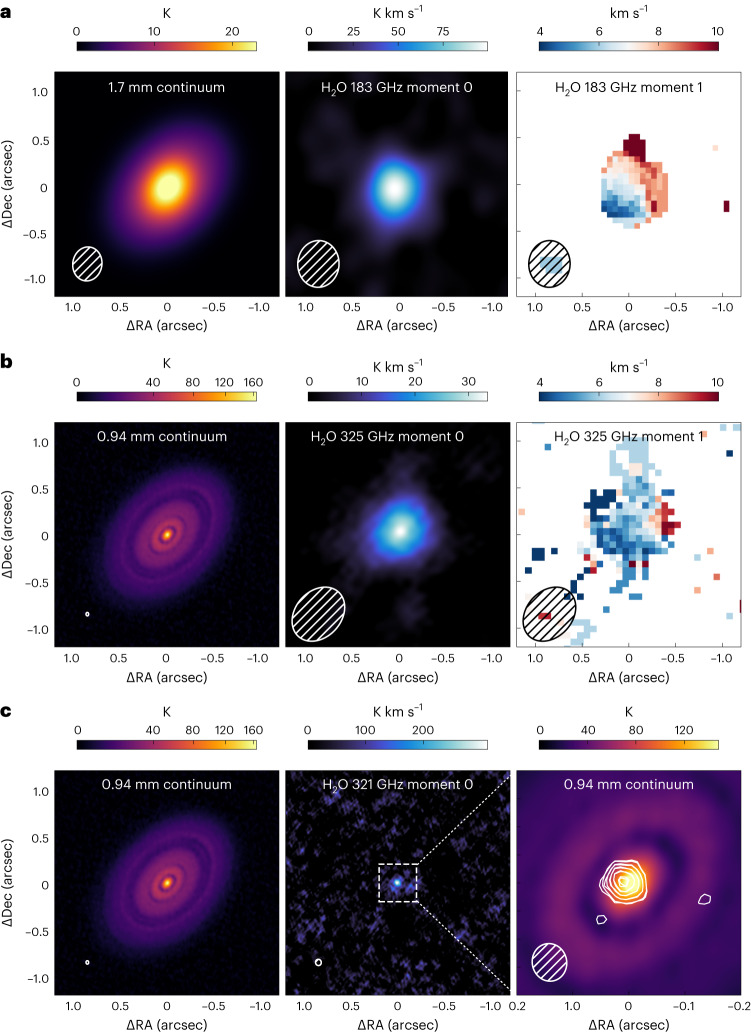
Continuum intensity and water vapour moment maps. **a**, Left, 1.7 mm continuum image of HL Tau. Centre, integrated intensity map of the 183 GHz water line. Right, intensity-weighted velocity map of the 183 GHz water line after 4*σ* clipping on individual channels, where disk rotation is clearly detected. **b**, Same as **a**, for the 0.94 mm continuum and the 325 GHz water line. The intensity-weighted velocity map in this case is computed after 3*σ* clipping. **c**, left and centre, same as **a**, for the 0.94 mm continuum and the 321 GHz water line. No moment 1 map is shown due to low SNR. Right, zoom-in of continuum intensity, with [4,5,6,7,8]*σ* contours of the 321 GHz line moment 0 map, with *σ* = 13.3 mJy beam^−1^ km s^−1^. The r.m.s. associated with the the integrated intensity maps of the 183 and 325 GHz lines are, respectively, 28.2 and 46.3 mJy beam^−1^ km s^−1^. Brightness intensity (K). Integrated intensity (K km s^−1^). Intensity weighted velocity along the line of sight (km s^−1^). Distance from the phase center in Right Ascension (Delta RA). Distance from the same center in Declination (Delta Dec).

After self-calibration, we imaged both the continuum and the continuum-subtracted lines with the CASA software^31^. The continuum images are shown in Fig. 1. All the H_2_O lines and the ${\mathrm{H}}_{2}^{18}{\mathrm{O}}$ line were imaged with CASA 6.2.1 with natural weighting, to maximize point source sensitivity. The 183 GHz line presents a synthesized beam of 0.500″ × 0.442″ (position angle (PA) −3.0°) and was imaged with a channel width of 0.8 km ^−1^. The resulting beam for the 325 GHz water line is 0.640″ × 0.491″ (PA −42.1°), with a 1 km s^−1^ channel spacing. For the high-excitation 321 GHz line, only one long baselines execution block was available, and the beam is 0.067″ × 0.061″ (PA 12.2°), with a 5 km s^−1^ channel spacing. The ${\mathrm{H}}_{2}^{18}{\mathrm{O}}$ line was imaged with several different channel spacings. To have a one-to-one comparison with the main isotopologue line, in this Article we show the results with a channel width of 1 km s^−1^. The resulting beam is 0.779″ × 0.626″ (PA −46.7°).

**Fig. 2:**
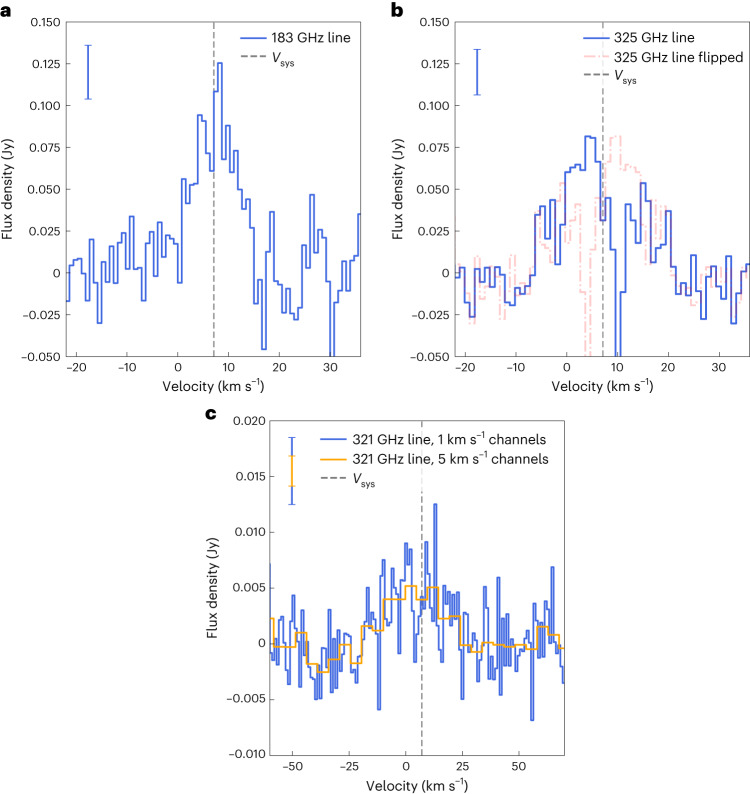
Water line spectra. **a**, Spectrum of the 183 GHz water line extracted over a circle with radius of 0.7″ centred on the continuum peak. **b**, Spectrum of the 325 GHz water line extracted over the same area, highlighting the mirrored (flipped) version of the spectrum with the dashed–dotted line. **c**, Spectrum of the 321 GHz water line extracted over a circle with radius of 0.06″ centred on the continuum peak. The velocity range on the *x* axis is different in **c**. The grey dashed line in all panels shows the systemic velocity $(V_{\rm{sys}})$ of 7.1 km s^−1^. In the top left of each panel, 2*σ* scale bars are reported for reference.

The spectrum of the two lowest excitation lines was extracted over a circular area with 0.7″ radius and is shown in Fig. 2. Both the 183 and 325 GHz lines are clearly detected across multiple channels, with the lines being centred on the systemic velocity of the system (7.1 km s^−1^; refs. ^32,33^). The 325 GHz line shows an absorption component at ~10 km s^−1^, as seen for other lines at similar upper energy levels^33^. The 183 GHz line shows a width of ~12 km s^−1^, while the higher-frequency lines show a slightly broader emission. The 183 GHz line spectrum exhibits a peak signal-to-noise ratio (SNR) of ~9.6, with a root mean square (r.m.s.) of 13.2 mJy over the flux density extraction area. The 325 GHz line spectrum instead shows a peak SNR of ~5.8, with a r.m.s. of 14.0 mJy. The higher-energy line at 321 GHz does not show a detection when integrating over the same area, due to the much smaller beam and the resulting high r.m.s. We thus extracted a spectrum over a circular area with radius 0.06″. The peak SNR is ~4.1, with a r.m.s. of 3.0 mJy. This transition shows the broadest line profile among the three detected lines, consistent with originating from the inner regions with higher Keplerian velocities. In all cases, the integrated intensity map shows a strong detection with a peak colocated with the dust continuum peak (Fig. 1). We obtain peak SNRs of 21.4, 19.8 and 8.1 in the integrated intensity maps of the 183, 325 and 321 GHz lines, respectively. The line fluxes extracted over a circular area centred on the continuum peak are reported in Table 1.

The intensity-weighted velocity maps (moment 1 maps) of the 183 and 325 GHz lines are shown in Fig. 1. In the high-SNR map of the 183 GHz line, disk rotation is clearly detected, with PA and systemic velocity in agreement with other brighter lines from the same dataset^33^, indicating that a displacement of the photocenter of the blue-shifted and red-shifted channels is detected at the current resolution.

**Extended Data Fig. 1:**
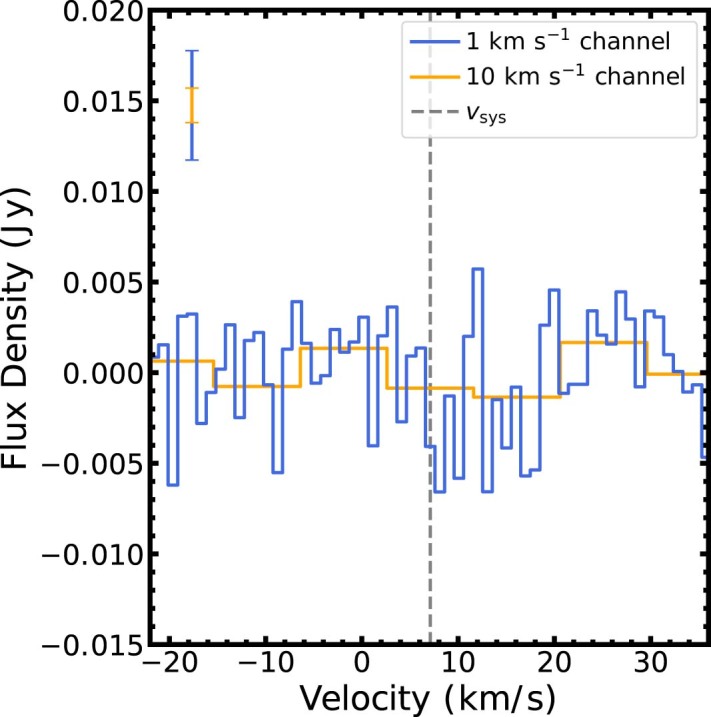
Non-detection of the 322 GHz ${\mathrm{H}}_{2}^{18}{\mathrm{O}}$ line. Spectrum of the 322 GHz ${\mathrm{H}}_{2}^{18}{\mathrm{O}}$ line, extracted over a circle with radius of 0. 7^*″*^ centered on the continuum peak, as for Fig. 2. Two spectra are shown, from channel maps with two different channel widths. The grey dashed line indicates the systemic velocity of 7.1 km s^−1^ ^32,33^. In the top left 2*σ* scale bars are reported for reference.

For the ${\mathrm{H}}_{2}^{18}{\mathrm{O}}$ line in band 7, we extracted the spectrum with the same methodology described for the three main isotopologue lines, over a 0.7″ radius circular area (Extended Data Fig. 1). A moment 0 map was computed over the same spectral range as the main isotopologue line. The line was not detected.

## The spatial distribution of water vapour

**Fig. 3:**
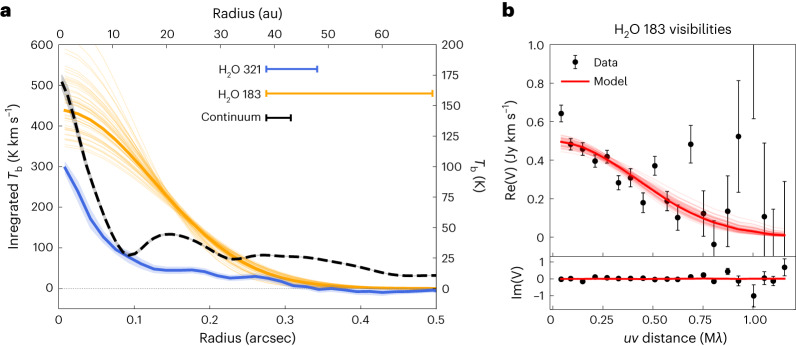
Spatial extent of water emission. **a**, Integrated brightness temperature (*T*_b_) radial profile of the 321 Hz line, reconstructed integrated *T*_b_ radial profile of the 183 GHz line and *T*_b_ profile of the 0.94 mm continuum emission. The thick orange line shows the best-fit model of the 183 GHz line assuming a Gaussian integrated intensity profile. Thin lines show randomly sampled realizations of the posterior distribution. The lines in the top right show the beam major axis. For the 183 GHz line, it portrays the smallest spatial scales to which the *uv* plane analysis is sensitive, which are ~2.3 smaller than the major axis of the respective natural beam. **b**, Real and imaginary part (Re and Im) of the recentred and deprojected visibilities (*V*) of the integrated intensity of the 183 GHz water line. The data points represent the weighted average of the visibilities within the 60-k*λ*-wide bins, with the scale bars showing the associated uncertainties (defined as $1/\sqrt{\left.\right(}{\sum }_{i}{w}_{i}\left.\right)$, with *w*_*i*_ being the weight of an individual visibility). The thick red line shows the best-fit model reproducing the profile shown in **a**. The units kλ| and Mλ| express distance in units of the observed wavelength λ|.

**Extended Data Fig. 2:**
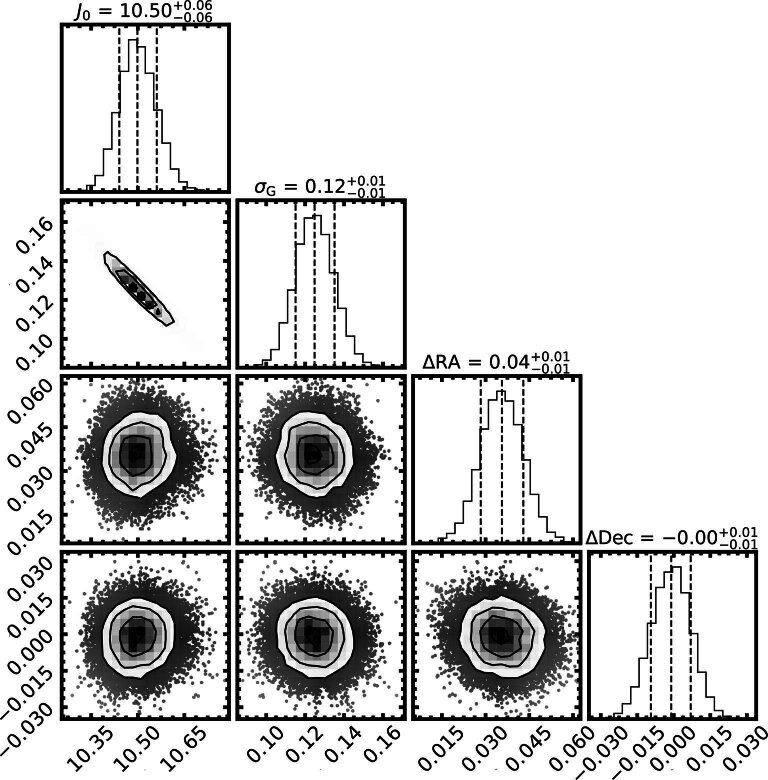
Corner plot of the radial extent of the 183 GHz line. Marginalized posterior distribution of the galario fit of the visibility points of the integrated intensity of the 183 GHz line (see Fig. 3). The dashed lines indicate the 16th, 50th and 84th percentiles of the marginalized posterior distributions.

The integrated intensity morphologies of the 183 and 321 GHz lines are remarkably different (Fig. 1), but so are the angular resolutions of the two moment maps. In order to derive radial profiles of the respective integrated intensities, we used two different approaches. First we focused on the highest-SNR line of the sample (at 183 GHz), which is also the coldest and therefore expected to trace the largest spatial extent. We averaged in frequency the interferometric data of the continuum-subtracted line in the same frequency range used to compute the moment 0 map. We then fitted the line integrated intensity visibilities with a simple Gaussian model using the galario package^34^ after fixing the inclination and PA to 46.7 and 138.0°, respectively, as derived from high-angular-resolution continuum observations^24^. The fit converges well to a Gaussian with *σ*_G_ = 0.12 ± 0.01″ (Fig. 3 and Extended Data Fig. 2; *σ*_G_ is the standard deviation of the Gaussian function). At a distance of 140 pc, this corresponds to *σ*_G_ = 16.8 ± 1.4 au. For the 321 GHz line; the much higher angular resolution allowed us to compute the radial profile of the integrated intensity by azimuthally averaging the moment zero map after deprojecting it by the known inclination (using the GoFish package^35^).

**Extended Data Fig. 3:**
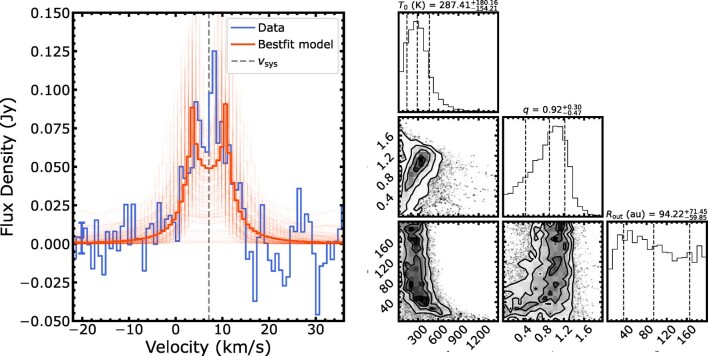
Fit of the 183 GHz line spectrum. Left: spectrum of the 183 GHz H_2_O line, as in Fig. 2. The uncertainty associated to each data point is shown in the left part of the spectrum as an errorbar. A random sampling of 100 profiles from the posterior distribution of the MCMC fit is shown, with the dark red line indicating the bestfit model. Right: marginalized posterior distribution of the fitted parameters, where no constraint can be retrieved on *R*_out_ from this analysis.

Figure 3 compares the two integrated intensity profiles with the band 7 azimuthally averaged continuum intensity profile. Both line profiles are clearly centrally peaked, indicating that the water vapour emission must originate above the optically thick continuum from the disk midplane. The lower excitation line is substantially more extended, showing that the water emission has a radially decreasing temperature (excitation) and that the high-excitation line is not optically thick outside the central beam. The same line shows detectable emission out to 0.3″ when boosting the SNR by azimuthally averaging. The 183 GHz temperature gradient is confirmed by fitting the high-SNR spectrum with a Keplerian disk model with a brightness temperature gradient, assuming that the line is close to being optically thick (Extended Data Fig. 3). Declining temperature profiles are preferred to flat ones, in agreement with the derived integrated intensity profile.

The warm brightness temperature profile of the 183 GHz line, which far exceeds the midplane temperatures obtained by analysis of multiwavelength continuum data^36^, indicates that the water vapour we are tracing originates in the warm disk upper layers. This is further supported by the peak of the 321 GHz emission, which is slightly shifted from the continuum peak (Fig. 1, bottom-right panel). Even though the two are consistent within the astrometric precision of the data, the apparent shift agrees with tracing water vapour on the side of the disk closer to the observer, which is in the northeast^25^, unocculted by the optically thick dust continuum. These findings set a stringent upper limit on the radius of the water snowline at 17 au. A more accurate determination will require simultaneous forward-modelling of the radial and spectral profiles of all three (sub)millimetre lines with the aid of thermochemical codes.

## Water column density

From the three main water lines, we computed the rotational diagram, under the assumption of optically thin emission and uniform excitation temperature across the energy range. While the very inner regions of the line emission are likely opaque, the bulk of the emission from the three lines cannot be optically thick, since this would imply a flux ratio that is equal to the square of the frequency ratio (in the Rayleigh–Jeans regime and local thermodynamical equilibrium), which we do not observe. While masing cannot be excluded as partly contributing to the emission, in the high-spectral-resolution spectra of the 183 and 325 GHz lines we have not identified individual narrow spectral features, which are in general a good signature for maser action together with high flux density. From the high densities of the inner disk, masing is not expected for the three lines analysed here, and the only contribution could originate in the very upper layers at low volume densities where collisional quenching of the masing action is less probable. In the rotational diagram, we used degeneracy quantum numbers that account for a 3:1 ortho-to-para ratio, and a partition function that considers all states within the same assumption^37^. No rescaling of the ortho- and para-line fluxes is needed to compute the total water column. We accounted for 10% absolute flux systematic uncertainties in the line fluxes.

**Fig. 4:**
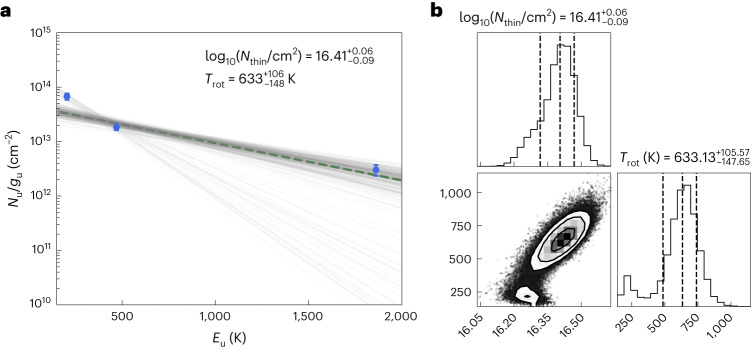
Rotational diagram. **a**, Rotational diagram of the three water lines, with line fluxes extracted as in the text. The fit does not lead to a unique solution, indicating that the assumption of uniform temperature is inadequate. **b**, Posterior distribution of the rotational diagram fit. The dashed lines indicate the 16th, 50th and 84th percentiles of the marginalized posterior distributions.

**Extended Data Fig. 4:**
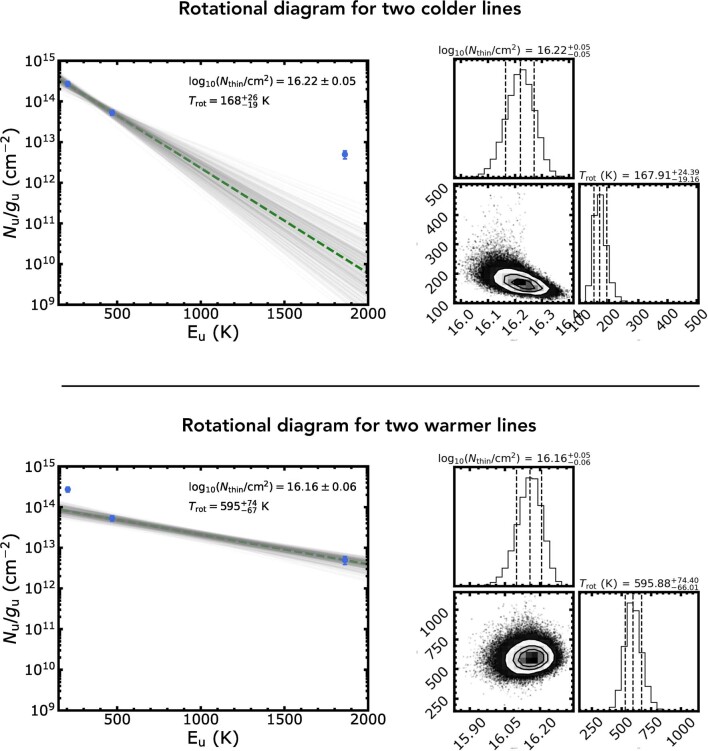
Rotational diagrams of line pairs. Rotational diagrams and marginalized posterior distribution of the MCMC exploration for individual fits on the two lowest and two highest energy lines, respectively. The green dashed line in the left panels shows the best fit. The black dashed lines in the corner plots indicate the 16th, 50th and 84th percentiles of the marginalized posterior distributions.

The rotational diagram does not provide a unique solution for the rotation temperature, as shown in Fig. 4. The Monte Carlo Markov chain (MCMC) exploration individuates two distinct temperatures, which realistically indicate a continuous gradient in the excitation temperature of the water vapour. Fitting either the two lower-energy lines or the two higher-energy lines separately, the rotational diagram indicates excitation temperatures of $21{4}_{-29}^{+42}$ and $78{9}_{-110}^{+127}$ K, respectively, in line with the two classes of solutions obtained in the joint fit (Extended Data Fig. 4). The lower-temperature solution is sensitive to colder water vapour in the range of expected desorption temperatures of water ice in space, suggesting that the bulk of the water emission from the 183 GHz line traces water gas in the proximity of the snow surface. The higher-temperature solution is driven by warm gas in the upper layers of the terrestrial planet-forming regions of the disk, which are well imaged by the high-resolution 321 GHz line integrated intensity map, and likely by the lines being close to be optically thick. The excitation temperature of the warm gas is in broad agreement with mid-infrared line fluxes in the 12.37–12.41 μm range on the same source (in particular, the o-H_2_O 16_4 13_–15_1 14_ line with upper state energy *E*_up_ = 4,948 K (ref. ^27^)). These high temperatures allow for water vapour formed in situ via gas-phase reactions^38,39^.

All the rotational diagrams robustly constrain the column density of water gas (in the optically thin limit and above the optically thick continuum). The joint fit shows a total water column density ${\log }_{10}({N}_{{{{\rm{thin}}}}}/{{{{\rm{cm}^2}}}})=16.4{1}_{-0.09}^{+0.06}$ within 0.7″ (~100 au) from the star. This corresponds to ~3.7 Earth oceans (7.1 × 10^−2^ lunar masses) of water vapour. Given the optically thin assumption, this has to be considered a tight lower limit. Since most of the emission originates from ≲0.12″ (17 au), assuming that the entirety of it is confined within this radial range, we obtain an average column density of ${\log }_{10}({N}_{{{{\rm{thin}}}}}/{{{{\rm{cm}^2}}}})\approx18.1{0}_{-0.09}^{+0.06}$, when accounting for the disk inclination.

The non-detection of the ${\mathrm{H}}_{2}^{18}{\mathrm{O}}$ line can determine an upper limit of the average optical depth expected for the H_2_O 325 GHz line (${\tau }_{{{{{\rm{H}}}}}_{2}{{{\rm{O}}}}}$). Assuming that ${\tau }_{{{{{\rm{H}}}}}_{2}{{{\rm{O}}}}}=530\,{\tau }_{{{{{\rm{H}}}}}_{2}^{18}{{{\rm{O}}}}}$ (using the oxygen isotope ratio measured in the solar wind^40^), we obtain that ${\tau }_{{{{{\rm{H}}}}}_{2}{{{\rm{O}}}}} < 14$. By turning the argument around, the optically thin assumption for the 325 GHz line provides an upper limit of *N*(H_2_O)/*N*(${\mathrm{H}}_{2}^{18}{\mathrm{O}}$) = 40 in HL Tau, well in agreement with oxygen fractionation levels in our own solar system^41^. The non-detection of ${\mathrm{H}}_{2}^{18}{\mathrm{O}}$ shows that observational campaigns aimed at targeting water in inner disks of quiescent disks with ALMA should privilege the main isotopologue as a first choice, with follow-up observations of robust detections targeting HDO and ${\mathrm{H}}_{2}^{18}{\mathrm{O}}$ to derive more accurate column densities and set stringent limits on the water deuteration^19^.

Assuming that the bulk of the water emission originates from ≲17 au from the star, we can compare the total mass of water (${M}_{{{{{\rm{H}}}}}_{2}{{{\rm{O}}}}}$) to the mass of dust *M*_dust_ estimated from multiwavelength continuum analysis of ALMA and Karl G. Jansky Very Large Array data in the same radial range^36^. By using the dust surface density from this study, we obtain *M*_dust_ ≈ 13*M*_Earth_, which leads to a water-to-dust mass ratio of ${M}_{{{{{\rm{H}}}}}_{2}{{{\rm{O}}}}}/{M}_{{{{\rm{dust}}}}}\approx6\times 1{0}^{-5}$. This number is much lower than the expected water abundance in inner disks (water-to-dust mass ratio ~10^−2^). The optical thickness of water can only marginally alleviate the problem, given the non-detection of ${\mathrm{H}}_{2}^{18}{\mathrm{O}}$. Continuum subtraction could also marginally reduce the water brightness temperature^42^ but in this case is not expected to reduce the water fluxes by more than a factor of 2 (see Fig. 3). The low water-to-dust ratio further strengthens the interpretation that with ALMA, we are probing only the upper layers of the disk, above the optically thick screen of the dust continuum emission, which shows optical depths between ~5 and 10 at 0.9 mm within the inner 17 au (ref. ^36^). The large cavity seen in the HDO and ${\mathrm{H}}_{2}^{18}{\mathrm{O}}$ integrated intensity maps of V883 Ori (ref. ^19^) further supports that the observation of a large column of water is hindered by the optically thick continuum in the inner disk.

## Conclusions

These new ALMA data reveal high-significance detections of three distinct rotational transitions of the main isotopologue of water vapour in the inner regions of the ringed HL Tau disk. These observations pave the way to the characterization of the water content of the inner regions of protoplanetary disks. The tremendous angular resolution and sensitivity of the ALMA telescope, even in spectral ranges of low atmospheric transmission, are providing spatially and spectrally resolved images of the vapour of the main water isotopologue in a planet-forming disk. Analyses of the morphology of the water emission, the spectrum of the highest SNR line and the excitation conditions jointly indicate that the (sub)millimetre lines are probing warm gas in the disk upper layers above the water snow surface, with a radially decreasing temperature profile. The non-detection of ${\mathrm{H}}_{2}^{18}{\mathrm{O}}$ and the low water-to-dust ratio in the inner 17 au show that the observations are probing marginally optically thick gas above the opaque dust continuum emission. These results highlight that the water content of quiescent protoplanetary disks at (sub)millimetre wavelengths is most efficiently unveiled by targeting the main isotopologue, in particular in disks with high-continuum optical depths within the water snowline.

## Methods

### Observations, data reduction and imaging

HL Tau was observed in both band 5 and band 7 with the ALMA program 2017.1.01178.S (principal investigator: E.H.), targeting the two para-water lines at 183.31004 and 325.15297 GHz, respectively. HL Tau was also observed in band 7 to target the latter water line with program 2022.1.00905.S (principal investigator: S.F.), together with the ${\mathrm{H}}_{2}^{18}{\mathrm{O}}$ line at 322.46517 GHz (see Table 1). The molecular coefficients for the three transitions are reported in Tables 1 and 3.

**Table 3:** Molecular coefficients of the observed H_2_O isotopologue transitions

Transition	*g*_u_	*A*_ul_ (s^−1^)	*Q* (100 K)	*Q* (200 K)	*Q* (300 K)	*Q* (400 K)
p-H_2_O 3_13_–2_20_	7	3.59 × 10^−6^	35.1	97.4	178.1	274.6
p-H_2_O 5_15_–4_22_	11	1.15 × 10^−5^	–	–	–	–
o-H_2_O 10_29_–9_36_	63	6.21 × 10^−6^	–	–	–	–
p-${\mathrm{H}}_{2}^{18}{\mathrm{O}}$ 5_15_–4_22_	11	1.05 × 10^−5^	–	–	–	–

Values are from the Jet Propulsion Laboratory database^51^, with radiative coefficients from ref. ^52^ and the updated partition function *Q* from the ExoMol database^37^, which well agrees with the one by ref. ^52^ in the temperature range explored in this Article. Only some representative values are reported in this table. The degeneracy quantum number of the ortho-state assumes an ortho-to-para ratio of 3. The partition function is computed over all states with the same ortho-to-para ratio.

In the 2017.1.01178.S ALMA program, the source was observed in band 5 on 21 September 2018 for a total integration time of 46 min, with 43 antennas and baselines ranging between 15 m and 1.4 km. The weather conditions during the observation were exceptional, with a median precipitable water vapour (PWV) column during the observations of ~0.19 mm. J0423-0120 was used as amplitude and bandpass calibrator and J0510+1800 for phase referencing. The band 5 spectral setup had six spectral windows (SPWs), five of which targeted different molecular transitions, including the 183 GHz water line, and a 1.875-GHz-wide spw for continuum observations at 170.004 GHz. In the same program, HL Tau was observed in band 7 in two spectral setups. The first was on 12 August 2019 with a time on-source of 31 min, with 48 antennas and baselines ranging between 41 m and 3.6 km. The median PWV column was 0.4 mm. J0431+1731 was used to cross-calibrate phases, and J0538-4405 for amplitude and spectral response. This first spectral setup consisted of four SPWs in frequency division mode: three with a maximum bandwidth of 1.875 GHz, and one with 1,920 244 kHz channels, targeting the water 325 GHz line. The second spectral setup was used in the same program with an execution block observed on 24 November 2017. The median PWV was 0.5 mm. J0519-4546 was used as amplitude and bandpass calibrator, and J0440+1437 as phase calibrator. The observation spent 33 min on the science target with 49 antennas, with a maximum baseline of 8,500 m. The spectral setup consisted of four SPWs in frequency division mode: three with maximum bandwidth, and one with 1,920 244 kHz channels, targeting the water 321 GHz line.

Finally, new data were taken in October 2022 with a more compact array in band 7 with program 2022.1.00905.S, with baselines ranging between 15 and 500 m and a total on-source time of 100 min with 41/45 antennas (in two execution blocks). The median PWV column was ~0.3. J0423-0120 was used for flux and bandpass calibration, while J0431+1731 was used for phase referencing. The setup had four SPWs, one centred on the 325.15 GHz water line and another targeting the ${\mathrm{H}}_{2}^{18}{\mathrm{O}}$ line at 322.46517 GHz.

The cycle 6 (9) data were calibrated by the ALMA pipeline using CASA v.5.4 (v.6.4)^31^. For the band 7 data, we combined the data from the two cycles. For both bands, we first self-calibrated each of the three execution blocks in phase, combining all spectral windows after flagging spectral ranges associated to line emission, and combining all scans. Following ref. ^43^, we then aligned the data by fitting a Gaussian to the continuum and shifting the phase centre to the continuum maximum with the fixvis and fixplanet tasks. We then self-calibrated the two short-baseline (in the case of band 5, only one set of baselines is available) execution blocks in both phase and amplitude, reaching a peak SNR of 9,950 (a ~500% improvement). In the case of band 7, we then combined the long-baseline execution block from the cycle 6 data and self-calibrated the data again in both phase and amplitude, reaching a peak SNR of 5,300 (a 210% improvement). We took particular care with the amplitude calibration, where the gain solution with scan-length intervals greatly improved the data quality. The models for self-calibration were constructed with CLEAN with Briggs weighting (robust = 0.5). The gain solutions were then applied to the full spectral data.

The band 5 continuum data were imaged with robust = 0.0. With a synthesized beam of 0.364″ × 0.312″ (PA −9.5°), the band 5 (1.70 mm) continuum presents a r.m.s. of 33 μJy, with a peak SNR of 2,457. The band 7 (0.94 mm) data were imaged with robust = −1.0. The data exhibit a r.m.s. of 36 y, with a peak SNR of 403 over a synthesized beam of 0.037″ × 0.029″ (PA 1.5°). A flux density for both images was obtained over an elliptical area with a semimajor axis of 1.3″ and semiminor axis and PA to trace the disk inclination and PA (46.7 and 138.0°, respectively^24^). Without accounting for absolute flux calibration uncertainties, we obtain a flux density of 323.0 ± 1.4 mJy and 1,677.7 ± 0.6 mJy at 1.70 and 0.94 mm, respectively, where the uncertainties have been computed as standard deviations of randomly selected masks with the same area of flux density extraction over emission-free regions of the continuum maps.

While the water lines have been imaged with natural weighting (see main text), several additional attempts with a range of UV tapers were performed to increase the sensitivity to extended emission, but they did not show any feature undetected with natural weighting. Both integrated intensity (moment 0) and intensity-weighted velocity (moment 1) maps were generated for the water lines. Moment 0 maps were computed by integrating channels between −2 and 16 km s^−1^ without any clipping (Fig. 1) for the band 5 line, between −6.4 and 20.6 km s^−1^ for the 325 GHz line and between −10.4 and 24.6 km s^−1^ for the 321 GHz line. For the two brighter lines, we integrated the moment 0 maps over a circular area centred over the emission peak and a radius of 0.7″. We obtained line fluxes of 973 ± 89 mJy km s^−1^ and 929 ± 89 mJy km s^−1^ (for the 183 and 325 GHz lines, respectively). Since the 325 GHz line shows an absorption red-shifted component, we also computed the underlying flux by considering the blue-shifted side only and multiplying it by a factor of 2. The resulting flux is 1,332 ± 89 mJy km s^−1^. The uncertainties on the line fluxes were computed by bootstrapping over 100 circular apertures in emission-free regions of the map and do not account for absolute flux calibration uncertainties. The same operation was applied to the 321 GHz line using a smaller 0.28″ radius extraction area. The resulting flux is 679 ± 135 mJy km s^−1^. The flux is much lower than the tentative detection by ref. ^20^ with the Submillimeter Array, where, however, the line is shifted by 30 km s^−1^ from the systemic velocity, indicating that the proposed emission may be originating from a large-scale flow that we filter out in our high-resolution data. For the 183 and 325 GHz lines, intensity-weighted velocity maps were generated using the bettermoments package^44^ after applying 4*σ* and 3*σ* clipping to individual channels, respectively.

The non-detection of the ${\mathrm{H}}_{2}^{18}{\mathrm{O}}$ 322 GHz line is shown in Extended Data Fig. 1.

### Fitting of the 183 GHz line spectrum

Given the high SNR of the 183 GHz line, we fitted its spectrum by analytically computing model spectra of a geometrically thin Keplerian disk, similarly to ref. ^45^. To do so, we assumed that the peak brightness temperature of the line decreases with radius as a power law:

1 $$T(R)={T}_{0}{\left(\frac{R}{10{{{\,\rm{au}}}}}\right)}^{-q}.$$

For a given radius *R* and azimuth *ϕ*, we assumed that the line intensity follows a Gaussian distribution velocity:

2 $$I(R,\phi ,v)=\frac{{B}_{\nu }(T\,)}{{d}^{2}}\exp \left(-\frac{\mu {m}_{{{{\rm{H}}}}}{(v-{v}_{{{{\rm{K}}}},{{{\rm{proj}}}}})}^{2}}{2{k}_{{{{\rm{B}}}}}T}\right),$$

where *B*_*ν*_(*T*) is the Planck function at temperature *T*, *k*_B_ is the Boltzmann constant, *d* is the distance of HL Tau (140 pc), *μm*_H_ is the mass of the water molecule and the Keplerian velocity projected along the line of sight *v*_K,proj_ can be written as follows:

3 $${v}_{{{{\rm{K}}}},{{{\rm{proj}}}}}={\left(\frac{G{M}_{\odot }}{R}\right)}^{1/2}\sin i\cos \phi ,$$

where *i* is the source inclination (46.7°) and *G* is the gravitational constant. We considered an optically thick limit when assuming a thermal broadening with a kinetic temperature equalling the brightness temperature. The flux density of the line can then be computed at every velocity *v* by integrating across the whole disk:

4 $$F(v)=\cos i\int\nolimits_{0}^{2\uppi }\int\nolimits_{{R}_{{{{\rm{in}}}}}}^{{R}_{{{{\rm{out}}}}}}I(R,\phi ,v)R{\mathrm{d}}R{\mathrm{d}}\phi ,$$

where $\cos i$ accounts for the geometrical projection on the sky. We fixed *R*_in_ to 0.1 au (but the model is not sensitive to this value for reasonably small radii) and sampled the disk with 150 points in radius and 550 in azimuth. We then convolved the models with a Gaussian kernel with the same channel width as in the data and sampled them at the same velocities. We kept three free parameters in the fitting procedure: *T*_0_, *q* and *R*_out_. We fitted the spectrum shown in Fig. 2 with the emcee package using flat priors on the three free parameters: [10,1500] K, [0,3], [2,200] au, respectively. We used 30 walkers, 1,000 steps of burn-in and 1,000 additional steps to sample the posterior distribution. Extended Data Fig. 2 shows the best-fit model and 100 random draws extracted from the posterior distribution. While the fit does not manage to constrain the outer radius of the emission, we obtain ${T}_{0}=28{7}_{-154}^{+180}$ and $q=0.9{2}_{-0.47}^{+0.30}$. The fit highlights a negative brightness temperature gradient in the radial profile, as seen in the reconstructed integrated intensity profile (Fig. 3) and as hinted by the rotational diagram shown in Fig. 4.

### Parametric fit of the integrated intensity profile in the visibility plane

In order to extract the radial extent of the 183 GHz line, we performed a parametric fit of its integrated intensity radial profile in the visibility plane by exploiting the galario package. After averaging the continuum-subtracted visibilities in the same spectral range used to compute the moment 0 map, we fitted the visibility data by Fourier transforming a projected integrated intensity profile in the same *uv* points sampled during the observations. We modelled the radial profile with a simple Gaussian prescription

5 $$J(R)={J}_{0}\,\exp \left(-\frac{{R}^{2}}{2{\sigma }_{{{{\rm{G}}}}}^{2}}\right),$$

where we considered four free parameters: *J*_0_, *σ*_G_ and the disk centre (ΔRA and ΔDec). We fixed the inclination and PA to those obtained from high-resolution continuum imaging^24^. The fit was performed with the emcee package, where the *J*_0_ parameter was sampled in log space. We used the following flat priors on the parameters:

$$\begin{array}{l}{\log }_{10}({\,J}_{0}/{{{\rm{steradian}}}})\in [1,20],{\sigma }_{{{{\rm{G}}}}}\in {[0,1.5]}^{{\prime\prime} },\Delta {{{\rm{RA}}}}\in\\ {[-0.4,0.4]}^{{\prime\prime} },\Delta {{{\rm{dec.}}}}\in {[-0.4,0.4]}^{{\prime\prime} }.\end{array}$$

The posterior distribution was sampled with 50 walkers and 1,000 steps, after 1,000 steps of burn-in. The MCMC exploration of the posterior space converges well, as shown in Extended Data Fig. 3.

### Rotational diagram and optical depth constraints

To compute the rotational diagram of the water molecule, we use the same approach as in, for example, refs. ^46,47^. In the optically thin assumption, we can compute the column density *N*_thin_ and the rotational temperature *T*_rot_ by measuring the integrated flux *S*_*ν*_Δ*v*:

6 $${N}_{{{{\rm{thin}}}}}=\frac{4\uppi }{{A}_{{{{\rm{ul}}}}}hc}\frac{{S}_{\nu }\Delta v}{\varOmega }\frac{Q({T}_{{{{\rm{rot}}}}})}{{g}_{{{{\rm{u}}}}}}\,\exp \left(\frac{{E}_{{{{\rm{u}}}}}}{{T}_{{{{\rm{rot}}}}}}\right),$$

where *Ω* is the solid angle used for flux extraction (see previous section), *A*_ul_ is the Einstein coefficient of the considered transition, *h* and *c* are the Planck constant and the speed of light in vacuum, *Q* is the partition function, *E*_u_ is the upper state energy (in K) and *g*_u_ is the upper state degeneracy. Using the relation between *N*_u,thin_ and *N*_thin_, the same equation can be written in logarithmic form^48^:

7 $$\ln \frac{{N}_{{{{\rm{u}}}},{{{\rm{thin}}}}}}{{g}_{{{{\rm{u}}}}}}=\ln {N}_{{{{\rm{thin}}}}}-\ln Q({T}_{{{{\rm{rot}}}}})-{E}_{{{{\rm{u}}}}}/{T}_{{{{\rm{rot}}}}}.$$

We performed a linear regression using the emcee sampler^49^ in the $[\ln ({N}_{{{{\rm{u}}}},{{{\rm{thin}}}}}/{g}_{{{{\rm{u}}}}}),\,{E}_{{{{\rm{u}}}}}]$ space to extract *N*_thin_ and *T*_rot_. We used the molecular coefficients reported in Table 3. In the fitter, the partition function was determined with a cubic spline interpolation across the rotational temperatures listed in ref. ^37^. The same approach was used for the rotation diagram analysis with six to eight transitions of o- and p-water in evolved stars^50^. In the MCMC sampling, we used 128 walkers and 2,000 steps (after 1,000 steps of burn-in). While Fig. 4 shows the result and the marginalized posterior distributions of the fit of all three lines, Extended Data Fig. 4 portrays the individual fits on the two colder and warmer lines, respectively.

To compute the upper limit on the optical depth of the 325 GHz water line, we exploited the non-detection of the ${\mathrm{H}}_{2}^{18}{\mathrm{O}}$ line, which has almost identical molecular coefficients. Assuming that the column density of the main isotopologue line (${\tau }_{{{{{\rm{H}}}}}_{2}{{{\rm{O}}}}}$) is equal to $530\times {\tau }_{{{{{\rm{H}}}}}_{2}^{18}{{{\rm{O}}}}}$ (ref. ^40^), we can write

8 $$\frac{{({S}_{\nu }\Delta v)}_{{{{{\rm{H}}}}}_{2}{{{\rm{O}}}}}}{{({S}_{\nu }\Delta v)}_{{{{{\rm{H}}}}}_{2}^{18}{{{\rm{O}}}}}}\approx \frac{1-{\rm{e}}^{-{\tau }_{{{{{\rm{H}}}}}_{2}{{{\rm{O}}}}}}}{1-{\rm{e}}^{-{\tau }_{{{{{\rm{H}}}}}_{2}{{{\rm{O}}}}}/530}}.$$

Using the 3*σ* upper limit on the ${\mathrm{H}}_{2}^{18}{\mathrm{O}}$ transition and the measured flux of the H_2_O line (after correcting for absorption, since the absorbing column of ${\mathrm{H}}_{2}^{18}{\mathrm{O}}$ will have the same scaling factor of 530), a numerical solution of the equations leads to ${\tau }_{{{{{\rm{H}}}}}_{2}{{{\rm{O}}}}} < 14$, as reported in the main text.

## Data Availability

All the ALMA data are publicly available on the ALMA archive (https://almascience.nrao.edu/aq/), with program IDs 2017.1.01178.S and 2022.1.00905.S.

## Code availability

The Python packages used in the data analysis are all publicly available. The calibration and fitting scripts can be obtained from S.F. upon reasonable request.
